# Influenza A virus infection induces initial proliferation of commensal *Streptococcus pneumoniae* in the larynx leading to dissemination into the lower respiratory tract

**DOI:** 10.1128/jvi.00555-26

**Published:** 2026-06-29

**Authors:** Kohsuke Kato, Keekushan Okamura, Yuki Nakamura, Mana Iwata, Mikako Hirohama, Yukino Ogura, Masamitsu Kono, Muneki Hotomi, Tomoko Sumitomo, Atsushi Kawaguchi

**Affiliations:** 1 Department of Infection Biology, Institute of Medicine, University of Tsukuba 38515, Tsukuba, Japan; 2 Graduate School of Comprehensive Human Sciences, University of Tsukuba 13121, Tsukuba, Japan; 3 College of Medical Sciences, School of Medicine and Health Sciences, University of Tsukuba 13121, Tsukuba, Japan; 4 Department of Otolaryngology-Head and Neck Surgery, Wakayama Medical University 13145, Wakayama, Japan; 5 Department of Oral Microbiology, Graduate School of Biomedical Sciences, Tokushima University 13109, Tokushima, Japan; 6 Transborder Medical Research Center, University of Tsukuba 13121, Tsukuba, Japan; 7 Microbiology Research Center for Sustainability, University of Tsukuba 13121, Tsukuba, Japan; 8 Center for Quantum and Information Life Sciences, University of Tsukuba 13121, Tsukuba, Japan; Fred Hutchinson Cancer Center Vaccine and Infectious Disease Division, Seattle, Washington, USA

**Keywords:** secondary bacterial infection, influenza virus, *in vivo* imaging, animal model

## Abstract

Secondary bacterial pneumonia is a major complication of influenza A virus (IAV) infection, frequently caused by *Streptococcus pneumoniae*. Pneumococci asymptomatically colonize the upper respiratory tract by forming biofilms. Viral infections, such as IAV, disrupt this stable colonization, leading to bacterial migration to the lower respiratory tract, and the development of severe pneumonia. However, the specific organs and tissues where IAV infection enhances the replication capacity of pneumococci in the biofilm state remain unclear. In this study, we engineered a mutant *S. pneumoniae* strain carrying the near-infrared luciferase gene, *Akaluc*, which is selectively expressed during the log phase. Using this strain, we established a mouse model that enables *in vivo* imaging of pneumococcal activation following IAV infection. In this model, IAV infection resulted in a marked increase in luminescence signals in the larynx, accompanied by increased bacterial proliferation in the lower respiratory tract. These results suggest that the larynx serves as a key site for pneumococcal activation and transition from biofilm to planktonic state during secondary bacterial pneumonia. Our imaging approach offers a powerful tool for elucidating host-pathogen interactions *in vivo* and may contribute to the development of preventive strategies against secondary bacterial pneumonia following IAV infection.

**IMPORTANCE:**

Seasonal influenza virus infection increases the risk of severe bacterial pneumonia, but the underlying mechanisms remain unclear. In this study, we used a mouse model and a pneumococcal mutant strain expressing a near-infrared luminescent protein in the log phase, enabling *in vivo* monitoring of the enhanced replication of pneumococci in deep tissues. We discovered that following influenza virus infection, bacterial replication is predominantly activated in the larynx, indicating that the larynx serves as a key site for activation of commensal pneumococci. This insight may inform the development of better strategies to prevent secondary bacterial pneumonia.

## INTRODUCTION

Seasonal influenza A virus (IAV) infection is often complicated by secondary bacterial pneumonia, with *Streptococcus pneumoniae* being the most common causative agent (1). During the 1918 “Spanish flu” pandemic, over 95% of fatalities were attributed to secondary bacterial pneumonia caused by pneumococci (1, 2). Even in the latter half of the 20th century, after the introduction of antibiotics, secondary bacterial pneumonia remained a major cause of death following IAV infection. More recently, during the 2009 H1N1 pandemic, bacterial pneumonia was reported in 25%–50% of fatal cases (3).

*S. pneumoniae* is a Gram-positive facultative anaerobe that forms biofilms on the mucosal epithelium of the upper respiratory tract and colonizes without causing symptoms (4–6). However, viral infections or the inhalation of harmful substances can promote the migration of pneumococcus from the upper to the lower respiratory tract, leading to pneumonia or invasive pneumococcal diseases, such as bacteremia and meningitis (7). Over 100 pneumococcal serotypes have been identified, with variation in colonization capacity and invasiveness depending on factors such as capsular thickness, composition, and antigenicity (8–10). Once colonized in the upper respiratory tract—including the nasal cavity and nasopharynx—pneumococci enhance their adhesion to the airway epithelium and evade host immunity through biofilm formation (4). *In vitro* studies have demonstrated that pneumococci exhibit reduced proliferation within biofilms while activating the expression of genes involved in adhesion and biofilm maintenance (11–13). In contrast, pneumococci that disperse from the biofilm and shift to a planktonic state are thought to trigger secondary bacterial pneumonia by activating genes associated with virulence factors and migration to the lower respiratory tract (13, 14). However, it remains unclear which specific tissues or organs colonized by pneumococci serve as the primary sites for pneumococcal activation from the biofilm state upon IAV infection.

In recent years, *in vivo* imaging techniques utilizing luciferase-based bioluminescence have been widely employed to non-invasively visualize cellular dynamics and gene expression. This method enables longitudinal and real-time imaging of biological processes in specific tissues or cells expressing luciferase transgenes by administering a luciferin substrate. Typically, firefly luciferase and D-luciferin are used as the conventional bioluminescence system, emitting light at a peak wavelength of 562 nm. This luciferase-based *in vivo* imaging technology has been applied not only to mammalian cells but also to bacteria. However, due to substantial absorption of emitted light by water molecules and hemoglobin in the blood, luminescent signals are significantly attenuated, making the detection of deep-tissue signals particularly challenging.

To overcome the limitation of *in vivo* sensitivity, near-infrared (NIR) luciferin derivatives, such as AkaLumine, which are less affected by light absorption, and a mutant luciferase gene, *Akaluc*, optimized for high substrate specificity toward AkaLumine, have been developed (15, 16). In this study, we constructed an *S. pneumoniae* mutant strain in which the *Akaluc* gene is selectively expressed during the log phase, allowing us to visualize the activation of colonizing pneumococci from the biofilm state upon IAV infection *in vivo. In vivo* imaging of pneumococcus expressing Akaluc revealed that, following IAV infection, the pneumococcal activation predominantly occurs in the larynx. Our approach provides a valuable tool for understanding the spatial dynamics of pneumococcal activation *in vivo* and may contribute to the development of strategies to prevent secondary bacterial complications following IAV infection.

## RESULTS

### *Streptococcus pneumoniae* EF3030 strain efficiently colonized nasal cavity and proliferated in the lower respiratory tract upon IAV infection

**Fig 1:**
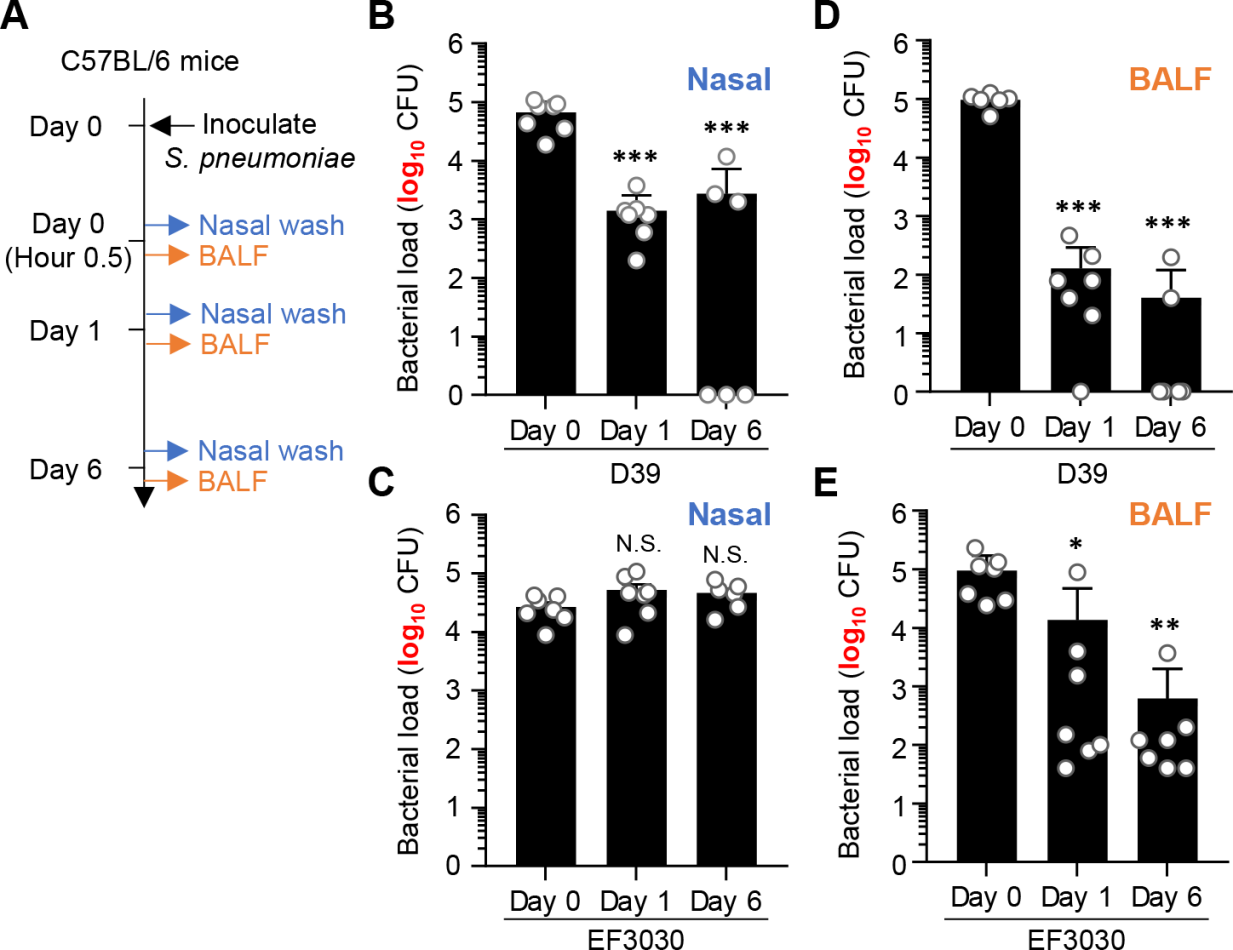
EF3030 strain stably colonizes the upper respiratory tract. (**A**) Experimental schema. C57BL/6 mice were intranasally inoculated with 1 × 10^6^ CFU of either D39 or EF3030 strain in a 15 μL volume. Bacterial loads in nasal washes and BALF were examined at 0, 1, and 6 days post-inoculation. (**B**) Bacterial loads of D39 in nasal washes (Day 0, *n* = 7; Day 1, *n* = 7; Day 6, *n* = 6). (**C**) Bacterial loads of EF3030 in nasal washes (Day 0, *n* = 7; Day 1, *n* = 7; Day 6, *n* = 6). (**D**) Bacterial loads of D39 in nasal washes (Day 0, *n* = 7; Day 1, *n* = 7; Day 6, *n* = 6). (**E**) Bacterial loads of EF3030 in BALF (Day 0, *n* = 7; Day 1, *n* = 7; Day 6, *n* = 6). *P < 0.05; **P < 0.01; ***P < 0.001; One-way ANOVA with Tukey’s test. Means ± SD are shown.

Bacterial strains capable of colonizing upper respiratory tract offer a more physiologically relevant model of bacterial dissemination into lower respiratory tract upon IAV infection (17) . Based on this rationale, we aimed to develop a mouse model for *in vivo* imaging of *S. pneumoniae* carrying a reporter gene. To select an *S. pneumoniae* strain that stably colonizes the upper respiratory tract as a commensal bacterium, we first examined the colonization efficiency of *S. pneumoniae* strains, D39 (serotype 2) and EF3030 (serotype 19F), in C57BL/6 mice. The D39 strain, a commonly used laboratory strain, is highly virulent with increased invasiveness (18). In contrast, the EF3030 strain, clinically isolated from a patient with otitis media, is less virulent and exhibits lower invasiveness (19, 20). Mice were intranasally inoculated with 1 × 10^6^ CFU of either strain, and bacterial loads in nasal washes and bronchoalveolar lavage fluids (BALF) were measured immediately after inoculation (day 0) and on days 1 and 6 (Fig. 1A). The nasal bacterial load of the D39 strain was significantly reduced within a day after inoculation (Fig. 1B), whereas the EF3030 strain persisted even at 6 days post-inoculation (Fig. 1C). This suggests that the EF3030 strain colonized the nasal cavity more efficiently than the D39 strain. However, the bacterial loads of both strains in BALF were barely detectable at 6 days post-inoculation (Fig. 1D and E). These results indicate that the EF3030 strain efficiently colonizes the nasal cavity but not the lower respiratory tract in mice without IAV infection.

**Fig 2:**
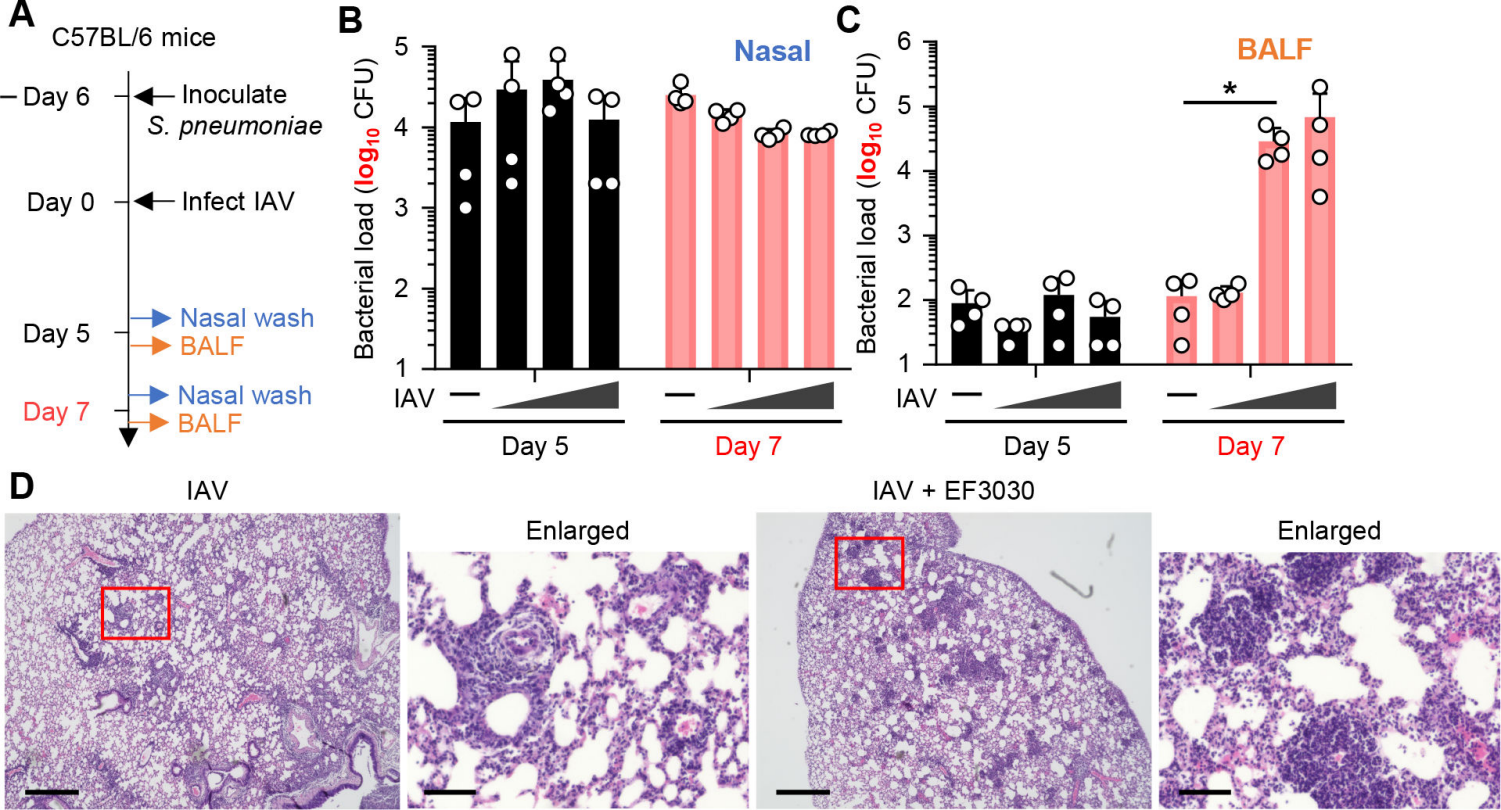
Enhanced proliferation of colonizing pneumococci by IAV infection. (**A**) Experimental schema. C57BL/6 mice colonized with EF3030 were intranasally infected with 15 μL of IAV at doses of 10, 30, or 100 PFU. Bacterial loads in nasal washes and BALF were examined at 5 and 7 days post-IAV infection. (**B**) bacterial loads in nasal washes (*n* = 4). (**C**) Bacterial loads in BALF (*n* = 4). *P < 0.05; two-tailed Student’s *t*-test. Means ± SD are shown. (**D**) Representative HE-stained images of lung tissues from C57BL/6 mice with or without EF3030 colonization at 7 days post-IAV infection. Scale bar, 500 μm; enlarged image, 100 μm.

We next examined whether the EF3030 strain, which colonizes the upper respiratory tract, can migrate to the lower respiratory tract upon IAV infection. Mice colonized with EF3030 were intranasally infected with 15 μL of A/Puerto Rico/8/1934 (PR8) at doses of 10, 30, or 100 PFU. At 5 and 7 days post-IAV infection, the bacterial loads in nasal washes and BALF were counted (Fig. 2A). At day 5, there was no significant difference in bacterial loads obtained from either nasal washes or BALF, irrespective of the virus titer (Fig. 2B and C). By day 7, the bacterial loads in BALF had significantly increased in an IAV dose-dependent manner (Fig. 2C), while those in nasal washes remained unchanged (Fig. 2B). No significant difference in body weight changes following IAV infection was observed between mice in which EF3030 had colonized and those in which it had not (Fig. S1). In contrast, histopathological analysis of HE-stained lung sections revealed marked tissue damage characterized by extensive disruption of alveolar structure (Fig. 2D). In the absence of EF3030, IAV infection induced dense mononuclear cell infiltration, primarily consisting of lymphocytes and macrophages, localized around the bronchioles (left panel). However, in EF3030-colonized mice, the inflammatory cell infiltration was more pronounced and organized into nodular aggregates that obscured the underlying alveolar structures, resembling bacterial pneumonia (right panel). These findings suggest that this EF3030-colonized mouse model may serve as a useful tool for studying the activation of nasal commensal bacteria upon IAV infection.

### Construction of EF3030 mutant strain expressing *Akaluc* gene

**Fig 3:**
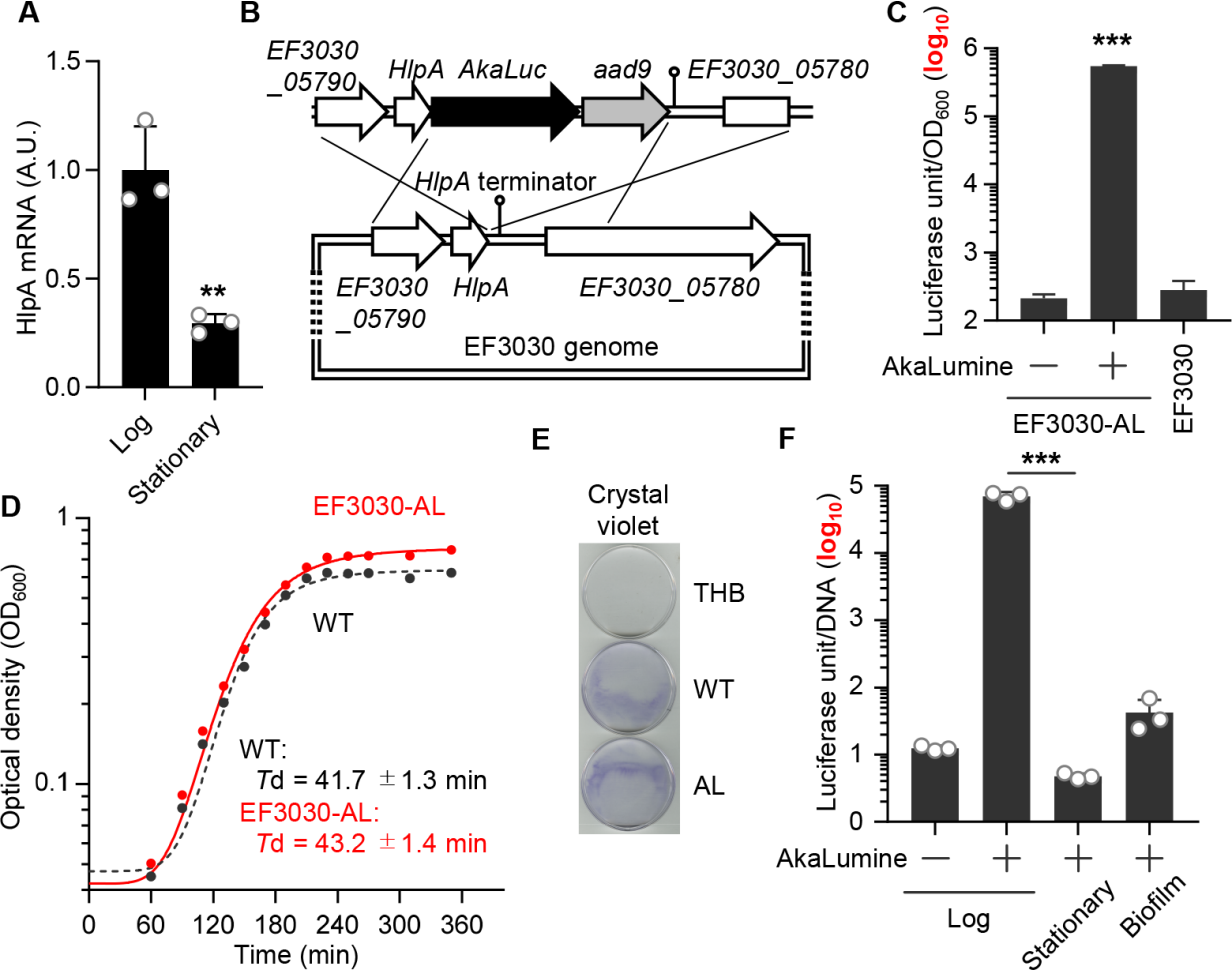
Construction of EF3030 mutant strain expressing the *Akaluc* gene. (**A**) The expression level of HlpA mRNA. Total RNAs were purified from *in vitro* culture of wild-type EF3030 strain at the log and the stationary phases (*n* = 3). The HlpA mRNA levels were quantified using RT-qPCR. The results were normalized to the level of 16S rRNA. **P < 0.01; two-tailed Student’s *t*-test. Means ± SD are shown. (**B**) DNA construction of the *Akaluc* gene downstream of the *HlpA* gene. A reporter unit consisting of the *Akaluc* gene and the spectinomycin resistance gene *aad9* was inserted downstream of the *HlpA* locus via homologous recombination. (**C**) Luciferase activity of the EF3030-AL strain. The EF3030 or EF3030-AL strain was incubated with 25 µM of AkaLumine-HCl at 37°C for 10 s, and the luciferase activity was measured. ***P < 0.001; two-tailed Student’s *t*-test. Means ± SD of luciferase activity normalized to OD_600_ values are shown. (**D**) Growth curve of the EF3030-AL strain. Bacterial growth of the EF3030 or EF3030-AL strain was monitored by measuring optical density at 600 nm (*n* = 3). The data were fitted using nonlinear regression with an exponential growth model. Doubling time (*T*d) was calculated based on the log phase of growth. Means ± SD are shown. (**E**) Biofilm formation activity of EF3030 and EF3030-AL strains. THB medium only (THB), EF3030 strain (WT), and EF3030-AL strain (AL) were anaerobically incubated at 37°C in non-treated polystyrene dishes, and then formed biofilm was stained with 0.1% crystal violet. (**F**) Luciferase activity of the EF3030-AL strain in log phase, stationary phase, and biofilm state. EF3030-AL strain in distinct growth phases was collected, and luciferase activity was measured. ***P < 0.001; two-tailed Student’s *t*-test. Means ± SD of luciferase activity normalized to 10 ng of bacterial genomic DNA are shown.

To examine the dynamics of commensal bacteria upon IAV infection *in vivo*, we constructed an EF3030 mutant strain expressing the *Akaluc* gene, a bioluminescent reporter that emits light from the red to near-infrared range, making it suitable for *in vivo* imaging in deep tissues. The histone-like protein HlpA is a DNA-binding protein of *S. pneumoniae* that functions to maintain the genome integrity (21). The transcriptional level of *HlpA* in the log phase was higher than that in the stationary phase in the wild-type EF3030 strain (Fig. 3A), suggesting that the expression of HlpA is associated with the proliferative rate of *S. pneumoniae*. Therefore, we inserted the *Akaluc* gene and the spectinomycin-resistance gene (*aad9*) downstream of the *HlpA* gene locus in a polycistronic manner via homologous recombination (Fig. 3B). The mutant EF3030 clone expressing *Akaluc* gene (EF3030-AL) showed higher luciferase activity in the presence of AkaLumine compared with the wild-type EF3030 strain (Fig. 3C). The growth rate of the EF3030-AL strain was not affected by the insertion of *Akaluc* gene (WT, doubling time (*T*d) = 41.7 ± 1.3 min; EF3030-AL, *T*d = 43.2 ± 1.4 min) (Fig. 3D). The *in vitro* biofilm formation activity of the EF3030-AL strain was comparable to that of wild-type EF3030 strain (Fig. 3E). Although a very strong luciferase activity was observed in the log state, no luciferase activity was detected during the stationary phase or in the biofilm state (Fig. 3F), suggesting that, by using the EF3030-AL strain, the luciferase activity specifically reflects the proliferative capacity of colonized pneumococci.

### *In vivo* imaging of mice colonized with the EF3030-AL strain upon IAV infection

**Fig 4:**
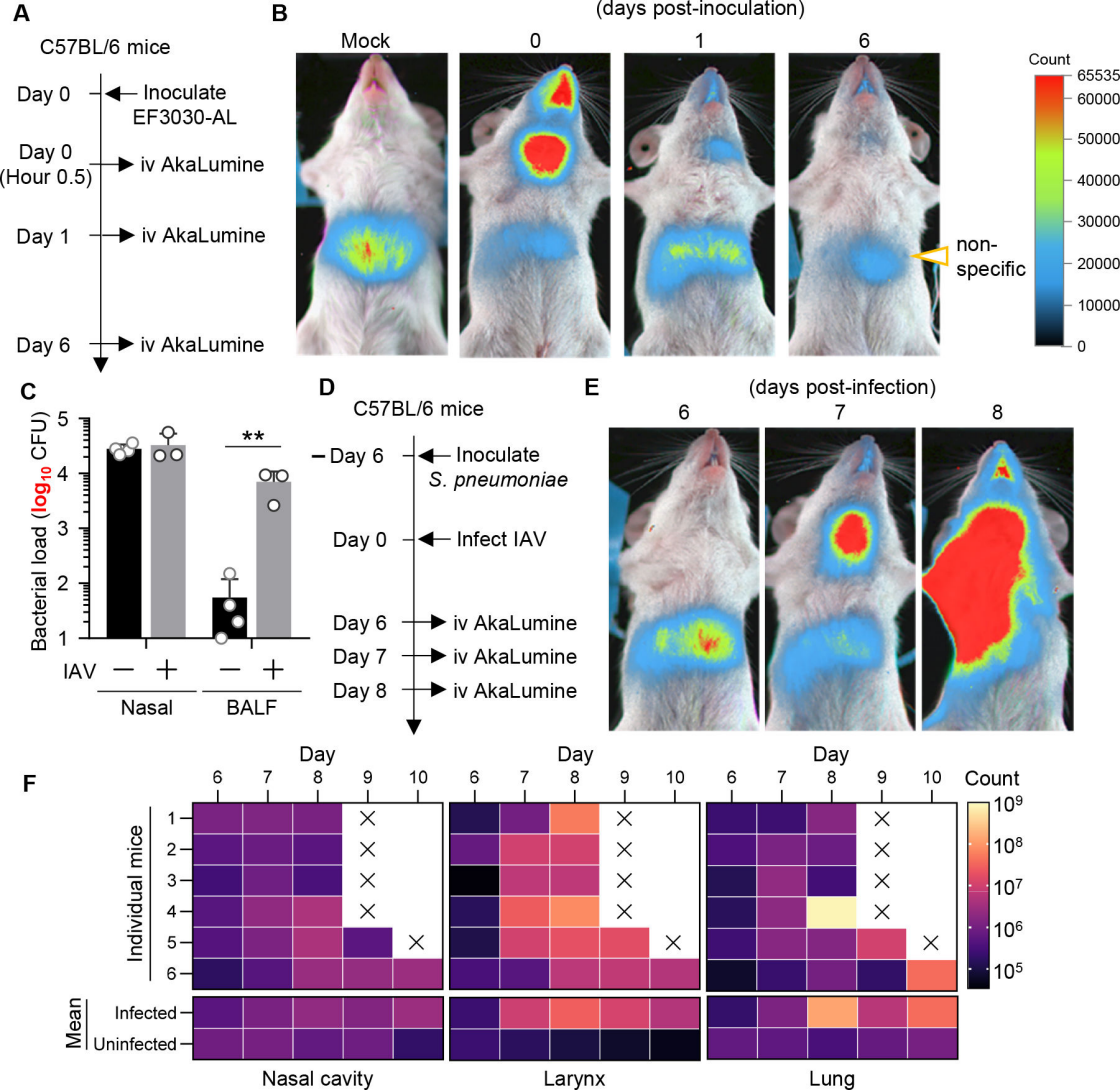
*In vivo* bioluminescence imaging of mice colonized with EF3030-AL strain. (**A and B**) *In vivo* bioluminescence imaging of EF3030-AL-colonized mice. C57BL/6 albino mice were intranasally inoculated with 1 × 10^6^ CFU of EF3030-AL in a 15 μL volume. *In vivo* bioluminescence imaging was performed at 0, 1, and 6 days post-inoculation following intravenous injection of 30 mM AkaLumine-HCl in a 50 μl volume (*n* = 6). A representative image from an EF3030-AL-colonized mouse is shown (**B**). (**C**) Bacterial loads in nasal washes and BALF were examined at 7 days post-IAV infection (uninfected, *n* = 4; infected, *n* = 3). **P < 0.01; two-tailed Student’s *t*-test. Means ± SD are shown. (**D–F**) *In vivo* bioluminescence imaging of EF3030-AL-colonized mice following IAV infection. EF3030-AL-colonized C57BL/6 albino mice were intranasally infected with IAV. *In vivo* bioluminescence imaging was performed at 6, 7, and 8 days post-IAV infection following intravenous injection of 30 mM AkaLumine-HCl in a 50 μL volume (*n* = 6). A representative image from an infected EF3030-AL-colonized mouse is shown (**E**). (**F**) Heatmap showing tissue-specific (nasal cavity, larynx, and lung) luminescence signals in individual mice from 6 to 10 days post-IAV infection. each row represents an individual IAV-infected mouse or the mean value for infected and uninfected groups (*n* = 6), respectively. The color scale indicates log_10_-transformed luminescence intensity. Cells marked with an “×” indicate animals that died prior to sample collection.

We next examined *in vivo* imaging of mice intranasally inoculated with 1 × 10^6^ CFU of EF3030-AL. Bioluminescent signals were monitored following intravenous injection of AkaLumine immediately after inoculation (referred to as day 0), and on days 1 and 6 (Fig. 4A and B). Strong luminescent signals were observed in the nasal cavity and pharyngolarynx immediately after bacterial inoculation (Fig. 4B). Although the bacterial load of EF3030 in nasal washes remained comparable on days 0, 1, and 6 (Fig. 1C and 4C), the luminescent signal in the nasal cavity markedly declined within 24 h (Fig. 4B). These results suggest that EF3030-AL stably colonized the nasal cavity likely in the stationary phase or in a biofilm state from day one post-inoculation (Fig. 3D). Notably, luminescence was also detected in the liver of mice that were not inoculated with EF3030-AL, likely due to non-specific metabolism of AkaLumine in the liver.

Next, EF3030-AL-colonized mice were intranasally infected with 100 PFU of IAV (Fig. 4D). At 6 days post-IAV infection, luminescent signals were primarily detected in the nasal cavity, remaining unchanged from those in uninfected mice. In contrast, strong luminescent signals emerged in the pharyngolarynx at 7 days post-IAV infection. Although some mice succumbed to IAV infection before bacterial dissemination to the lower respiratory tract, such dissemination was clearly observed in the remaining mice at 8 to 10 days post-IAV infection (Fig. 4E and F). These results suggest that the proliferative capacity of colonizing *S. pneumoniae* is enhanced around the pharyngolarynx upon IAV infection, followed by migration into the lower respiratory tract. Similar findings were observed not only with PR8 but also with the pdmH1N1 strain (A/California/07/2009) (Fig. S2).

**Fig 5:**
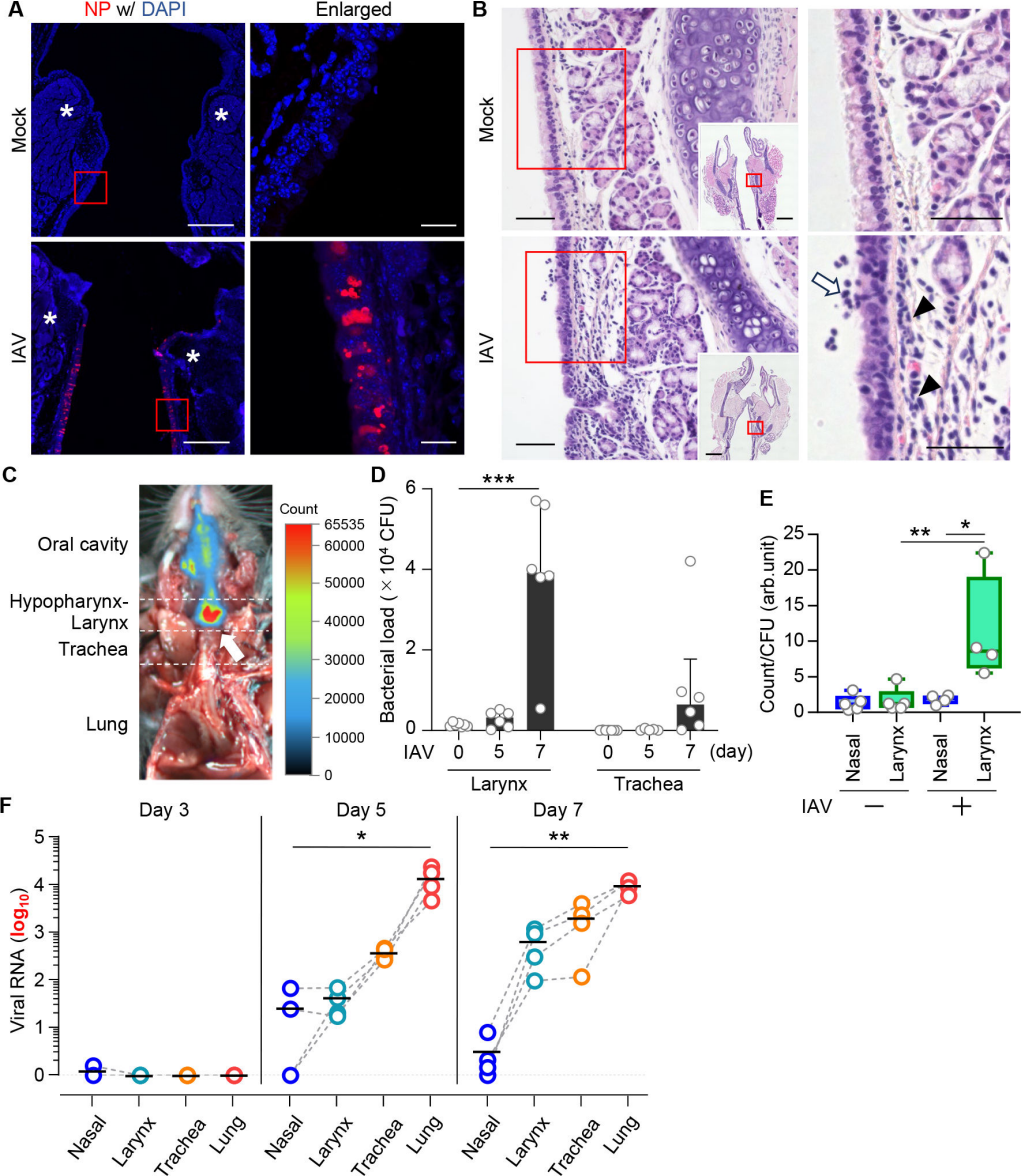
Enhanced proliferation of pneumococci in the larynx upon IAV infection. (**A and B**) C57BL/6 mice colonized with EF3030 were intranasally infected with 15 μL of IAV at a dose of 100 PFU. Immunofluorescence staining for nucleoprotein (**A**) and HE staining (**B**) of larynx sections were performed at 7 days post-infection. Representative images are shown. Scale bar, 500 μm; enlarged image 50 μm. DNA was counterstained with DAPI (blue). (**C**) *In vivo* bioluminescence imaging of EF3030-AL-colonized mice following IAV infection. EF3030-AL-colonized C57BL/6 albino mice were intranasally infected with IAV. At 7 days post-IAV Infection, mice were dissected after intravenous injection of 30 mM AkaLumine-HCl in a 50 μl volume. A representative image from an infected EF3030-AL-colonized mouse is shown. (**D**) Bacterial loads in the larynx and trachea. Pneumococci were collected from the larynx and trachea, and the bacterial loads were counted at 0, 5, and 7 days post-IAV infection (*n* = 6). **P < 0.01; two-tailed Student’s *t*-test. Means ± SD are shown. (**E**) At 7 days post-IAV infection, luminescent signal intensities were normalized to bacterial CFU counts in the nasal cavity and the larynx (*n* = 4). The center line indicates the median, the box limits indicate IQR (25th–75th percentiles), and the whiskers extend to 1.5 × IQR. **P < 0.01; *P < 0.05; one-way ANOVA with Tukey’s test. (**F**) Viral loads in the nasal cavity, larynx, trachea, and lungs. Total RNAs were purified from the nasal cavity, larynx, trachea, and lungs of EF3030-AL-colonized mice at 3, 5, and 7 days post-IAV infection (*n* = 4). The viral RNA levels were quantified using RT-qPCR. The results were normalized to the level of 18S rRNA. ***P < 0.001; **P < 0.01; *P < 0.05; Friedman test followed by Dunn’s multiple comparisons test. Means ± SD are shown.

In the tissues surrounding the pharyngolarynx, there are not only airway structures (such as pharynx, larynx, and trachea) but also glandular tissues, including the submandibular glands and lymph nodes. To define the site of viral infection, we performed histopathological analysis of the laryngeal sections from EF3030-colonized mice following IAV infection by IHC with anti-NP antibody (Fig. 5A) and hematoxylin and eosin (HE) staining (Fig. 5B). NP-positive signals were predominantly detected in the pseudostratified ciliated columnar epithelium in the subglottic region of the larynx (Fig. 5A). Furthermore, HE staining of the larynx demonstrated epithelial cell desquamation and accumulation of inflammatory cells within the lamina propria and submucosa (Fig. 5B). These findings indicate that the subglottic epithelium serves as a site of active viral infection and local inflammatory responses. In agreement with these observations, luminescent signals were mainly detected in the larynx (Fig. 5C).

To further examine the bacterial load at the larynx, EF3030-colonized mice were dissected at 5 or 7 days post-IAV infection, and the bacteria were collected by washing the larynx and trachea with PBS, respectively (Fig. 5D). In IAV-uninfected mice, a few thousand bacterial cells were detected in the larynx but not in the trachea, suggesting that a small number of *S. pneumoniae* also colonize the larynx (Fig. 5D), while the lower respiratory tract remains sterile in uninfected mice. In contrast, at 7 days post-IAV infection, the bacterial loads in the larynx increased more than 20-fold compared with uninfected mice. Notably, luminescence per CFU was significantly increased following IAV infection in the larynx but not the nasal cavity, suggesting enhanced bacterial proliferative capacity in the larynx (Fig. 5E).

To clarify the replication kinetics of IAV across airway tissues in our mouse model, we next quantified viral RNA levels in the nasal cavity, larynx, trachea, and lung by quantitative RT-PCR at 3, 5, and 7 days post-IAV infection (Fig. 5F). Viral RNA levels peaked in the lung at 5 days post-infection and subsequently increased in the larynx and trachea toward day 7, although remaining lower than those in the lung. These results indicate that, in our animal model, the enhanced growth of *S. pneumoniae* in the larynx is not simply dependent on the extent of local viral replication but rather reflects tissue-specific differences in colonization efficiency. Notably, as observed with PR8, similar findings were obtained with the pdmH1N1 strain (Fig. S2C). This strain has been reported to exhibit enhanced replication in the nasal cavity as previously reported (22) (Fig. S2D), indicating that the larynx supports pneumococcal expansion independent of viral tissue tropism in our model.

## DISCUSSION

In this study, we constructed an EF3030 mutant strain selectively expressing the *Akaluc* gene in the log phase (Fig. 3) and employed *in vivo* bioluminescence imaging to visualize the proliferative capacity of pneumococci colonizing the upper respiratory tract. Our results indicated that EF3030 predominantly colonizes the upper respiratory tract, particularly the nasal cavity (Fig. 1), with a smaller population present in the larynx (Fig. 5). After IAV infection, enhanced pneumococcal proliferation was observed in the larynx (Fig. 5), followed by migration and subsequent expansion in the lower respiratory tract (Fig. 2 and Fig. 4 and Fig. 5). Notably, the luminescent signal intensity remained relatively stable in the nasal cavity upon IAV infection (Fig. 5), suggesting that pneumococcal proliferation is predominantly enhanced in the larynx. Although the mechanisms underlying the enhanced proliferation of colonizing *S. pneumoniae* remain unclear, IAV infection may modulate pneumococcal gene expression and alter the commensal microbiota in the larynx. Previous studies have suggested that several host responses induced by IAV infection, such as hyperthermia, norepinephrine release, extracellular ATP released from damaged cells, and alterations in nutrient availability, may promote pneumococcal proliferation from the stationary phase or biofilm state (13, 14). Identification of host-derived molecules responsible for pneumococcal activation will be critical for understanding the mechanisms underlying IAV-commensal bacteria interactions.

The larynx is located at the anatomical boundary between the upper and lower respiratory tracts, possesses specialized structures such as cartilage, muscle, and vocal cords, and is exposed to various environmental factors, including microorganisms, air pollutants, and esophageal reflux (23). The laryngeal mucosa provides a multifaceted defense system comprising a mechanical epithelial barrier, the production of antimicrobial peptides, and a network of resident immune cells, including macrophages (24). Recent single-cell transcriptomic analysis has suggested that these local immune systems are dynamically regulated by interactions with commensal microbiota, which are essential for maintaining mucosal homeostasis and barrier integrity in the larynx (24). We found that IAV can infect the airway epithelium and induce the inflammatory cell infiltration in the larynx (Fig. 5A and B). IAV infection may disrupt this commensal-immune axis and compromise antimicrobial defenses, leading to the enhanced proliferation and dissemination of *S. pneumoniae* into the lower respiratory tract from the larynx. In addition to the enhanced proliferation of pneumococci, it is proposed that the expression of pneumococcal receptors, such as PAFR, pIgR, and GP96, is necessary for pneumococcal migration to the lower respiratory tract (5, 25, 26). Understanding how the expression of these genes is regulated by IAV infection in the larynx is also crucial for elucidating the molecular mechanisms underlying secondary bacterial infection following IAV infection.

*S. pneumoniae* is the primary pathogen responsible for secondary bacterial pneumonia following IAV infection. However, other bacterial species, such as *Staphylococcus aureus* and *Pseudomonas aeruginosa*, also cause secondary bacterial pneumonia. In the mouse larynx, *Streptococcus* is one of the dominant commensal genera (24). Nevertheless, commensal colonization by *Staphylococcus* and *Pseudomonas* species has also been observed (24). Future studies investigating whether other commensal bacterial species can similarly colonize the larynx and undergo proliferation following IAV infection will be important for broadening our understanding of the mechanisms underlying secondary bacterial pneumonia.

A limitation of this study is that we cannot entirely rule out the possibility that a small number of pneumococci, which may have remained in the lower respiratory tract, proliferate locally upon IAV infection, in addition to the migration of pneumococci from the larynx (Fig. 5). Future studies should focus on developing methods to specifically label pneumococci colonizing the larynx and track their dynamics *in vivo*. In this study, the EF3030 strain, which is a low-invasive *S. pneumoniae*, was used to stably colonize the upper respiratory tract (Fig. 1). Our mouse model successfully reproduced the enhanced proliferation of colonizing pneumococci upon IAV infection. Notably, histopathological analyses suggested that bacterial pneumonia was induced; however, the overall disease severity was dominated by viral pathology, which masked the contribution of pneumococcal virulence (Fig. S1). The severity of disease progression depends not only on the bacterial and viral strains but also on the mouse genetic background. Future studies should aim to optimize the conditions to better dissect the contribution of bacterial virulence and to extend these findings by examining additional pneumococcal strains that retain stable colonization in the upper respiratory tract while exhibiting higher virulence. Furthermore, seasonal IAV replication in humans is typically more prominent in the upper respiratory tract, including the nasal cavity, than in mice. Thus, the tissue distribution observed in our mouse model might not fully reflect that in humans. Nevertheless, laryngeal symptoms, such as hoarseness, are commonly observed in clinical settings, indicating involvement of the larynx during IAV infection. Our findings identify the larynx as a potential site for bacterial activation following IAV infection. Although the larynx may not be the sole or primary site of origin for secondary bacterial pneumonia, our data suggest that this region represents an underappreciated niche for pathogen interactions. Further studies using human samples or non-human primate models will be important to determine the translational relevance of these findings.

## MATERIALS AND METHODS

### Biological materials

Influenza A virus A/Puerto Rico/8/1934 was grown at 35.5°C for 48 h in allantoic sacs of 11-day-old embryonated eggs. The infected allantoic fluids were then collected and stored at −80°C. All viral infection experiments were conducted in a biosafety level 2 (BSL-2) facility at University of Tsukuba. *S. pneumoniae* D39 and EF3030 strains were grown at 37°C in Todd-Hewitt broth supplemented with 0.2% yeast extract (THY medium; BD Biosciences) or 5% sheep blood agar plates (Eiken Chemical). For the biofilm formation of *S. pneumoniae* EF3030 was grown at 37°C in Todd-Hewitt broth without yeast extract (THB medium; BD Biosciences).

### Construction of *S. pneumoniae* expressing *Akaluc* gene

A DNA construct encoding the Akaluc gene, optimized for codon usage in *S. pneumoniae*, and the downstream region of the *HlpA* gene, was chemically synthesized (Thermo Fisher). The 5′ homology arm, upstream of the *HlpA* gene from the RS05790 locus, and the aad9 gene with 3′ homology arm from the RS05780 locus were amplified by polymerase chain reaction (PCR) using the genomic DNA of the EF3030-sfGFP strain as a template. The primers used for amplification were as follows: 5′-CATATGTCCCATGCTCTTTCGGGACGGTAG-3′ and 5′-ATCTCTTTTGCCCAATCATCACTACCAGAATATGAAATTC-3′ for 5′ homology arm, and 5′-ACTCGTGGAACTTAGAGTGAGGAGGATATATTTGAATACATACG-3′ and 5′-TTTTGACCACCAGAAAGCTCAGCAATTTTCATCTG-3′ for 3′ homology arm. The homology arm regions and the HlpA-Akaluc sequence were then subjected to overlap PCR with the primers: 5′-CATATGTCCCATGCTCTTTCGGGACGGTAG-3′ and 5′-TTTTGACCACCAGAAAGCTCAGCAATTTTCATCTG-3′.

The EF3030 strain was cultured anaerobically at 37°C in THY medium containing 13 mM HCl and 0.05% glycine. After reaching OD_600_ = 0.1, the culture was diluted to OD_600_ = 0.03 with fresh THY medium and further supplemented with 4 mM NaOH, 0.2% BSA, 0.01% CaCl_2_, and 100 μg/mL competence-stimulating peptide (CSP) (27). After incubation at 37°C with 5% CO_2_ for 14 min, the culture was treated with the PCR-amplified donor DNA and incubated for 45 min at 37°C. The recombinant *S. pneumoniae* clones were isolated on blood agar plates containing 100 μg/mL spectinomycin.

### *In vitro* luciferase assay

The EF3030 or EF3030-AL strain was cultured anaerobically at 37°C in THY medium, and each bacterial culture was collected at OD_600_ = 0.35 (log phase) and 0.72 (stationary phase). For bacterial biofilm formation, the EF3030-AL strain was cultured anaerobically at 37°C in THY medium until the OD_600_ reached 0.3–0.5 (log phase). The bacterial culture was diluted with THY medium to adjust to 1 × 10^5^ CFU/mL, then 10 mL of culture was transferred into non-treated 10-cm polystyrene dishes and anaerobically incubated at 37°C for 18 h. After removing the medium, the dish was gently rinsed with PBS three times and stained with 0.1% crystal violet to confirm biofilm formation. Replicated dishes were also rinsed with PBS three times, and then the biofilm was collected by scraping with PBS. The biofilm pellet was resuspended with 100 μL of THY medium. Then, 25 μL of each bacterial culture was mixed with 2.5 μL of 250 μM AkaLumine-HCl, and the luciferase activity was measured by MiniLumat LB9506 (BERTHOLD). The genomic DNA was extracted from the remained bacterial cultures, and the DNA concentration was measured using NanoDrop spectrophotometer. The DNA amount was used as an internal control to normalize luciferase activity.

### Animal experiments

Ten- to 12-week-old male and female C57BL/6 Albino mice were purchased from The Jackson Laboratory. Wild-type EF3030 and EF3030-AL strains were anaerobically cultured in 10 mL of THY medium until the optical density at 600 nm (OD_600_) reached approximately 0.15–0.3 at 37°C. The cultures were centrifuged at 8,000 rpm for 3 min, and the pellets were washed with 10 mL of PBS. The bacterial pellet was then gently resuspended in PBS to a final concentration of 1 × 10^6^ CFU of *S. pneumoniae* per 15 μL. Mice were anesthetized by intraperitoneal administration of pentobarbital and then intranasally inoculated with 1 × 10^6^ CFU of *S. pneumoniae*. For IAV infection, *S. pneumoniae*-colonized mice were anesthetized by intraperitoneal administration of pentobarbital and then intranasally infected with 100 PFU of IAV in 15 μL of PBS.

For evaluation of bacterial load, the nasal washes were collected by flushing the nasal cavity twice with 1 mL of PBS. BALF was collected by washing the respiratory tract, from the trachea to the alveoli, twice with 2 mL of PBS. The larynx and trachea were washed with 1 ml of PBS, respectively. After centrifugation, the supernatants were removed, and bacterial pellets were spread onto 5% sheep blood agar plates and anaerobically incubated at 37°C overnight.

For *in vivo* imaging, the EF3030-AL-colonized mice were intravenously administered 50 μL of 30 mM AkaLumine hydrochloride (Fuji Film). The mice were analyzed using the Newton 7.0 *in vivo* imaging system (Vilber Bio Imaging) under isoflurane anesthesia for 30 min. The obtained images were analyzed using VILBER Lourmat software.

### Histological analyses

Mouse tissues were fixed with 10% neutral-buffered formalin for 48 h at room temperature and embedded in paraffin. Three-micrometer slices were subjected to hematoxylin and eosin (HE) and immunofluorescence staining with anti-NP antibody (28). Antigen retrieval was conducted by using citrate buffer (pH 6.0) in a pressure cooker for 5 min. The slices were blocked with PBS containing 3% BSA and 5% FBS for 30 min at room temperature. The primary antibody diluted in the blocking solution was incubated overnight at 4°C. HRP-conjugated secondary antibody was then incubated for 1 h at room temperature. TSA staining solution (PBS-T containing 0.0035% H_2_O_2_, 1% dextransulfate (SIGMA), 1 µg/mL Tyramide, and 50 ng/mL 4-iodophenol (TCI)) was incubated for 15 min at room temperature, protected from light. The images were obtained by using BZ-X810 (Keyence) or LSM700 (Zeiss).

### Quantitative real-time PCR

The wild-type EF3030 strain grown in THY medium was collected at the log phase (OD_600_ = 0.48) and the stationary phase (OD_600_ = 0.65). After treating with MAX Bacterial Enhancement Reagent (Ambion), total RNAs were isolated using the acid guanidium phenol chloroform method. cDNA was prepared from 200 ng of total RNA using ReverTraAce (Toyobo) with gene-specific reverse primers targeting *HlpA* and 16S rRNA, corresponding to those used for quantitative PCR. Mouse total RNAs were isolated from the nasal cavity, larynx, trachea, and lung using the acid guanidinium phenol chloroform method. cDNA was prepared from 1 μg of total RNA using ReverTraAce with gene-specific reverse primers targeting segment five viral RNA and 18S rRNA, corresponding to those used for quantitative PCR. Real-time PCR was carried out using SYBR Green Realtime PCR Master Mix-Plus (Roche) in the Thermal Cycler Dice Real-Time PCR system (TaKaRa). Primer sequences used in this study were as follows: 5′- ATGGCAAACAAACAAGATT-3′ and 5ʹ- TCACCAGCTGCAAGATAGT-3ʹ for *HlpA*; 5′- GGTGAGTAACGCGTAGGTAA-3′ and 5′-ACGATCCGAAAACCTTCTTC-3′ for 16S rRNA; 5′-GACGATGCAACGGCTGGTCTG-3′ and 5′-AGCATTGTTCCAACTCCTTT-3′ for segment five viral RNA; 5′-AACGGCTACCACATCCAAGG-3′ and 5′-GGGAGTGGGTAATTTGCGC-3′ for 18S rRNA.

### Statistical analysis

Statistical significance was determined by unpaired two-tailed Student’s *t*-test, one-way ANOVA with Tukey’s test, or Friedman test with Dunn’s multiple comparisons test using GraphPad Prism (version 7.03). N.S., not significant. ***P < 0.001, **P < 0.01, *P < 0.05.

## Data Availability

All data supporting the findings of this study are available within the main text and supplemental material.

## SUPPLEMENTAL MATERIAL

Supplemental figures jvi.00555-26-s0001.pdf Fig. S1 and S2.

ASM does not own the copyrights to Supplemental Material that may be linked to, or accessed through, an article. The authors have granted ASM a non-exclusive, world-wide license to publish the Supplemental Material files. Please contact the corresponding author directly for reuse.
