# Supplementary material for: Influenza A virus infection induces initial proliferation of commensal Streptococcus pneumoniae in the larynx leading to dissemination into the lower respiratory tract
Source: J Virol. 2026 Jun 29;100(7):e00555-26. doi: 10.1128/jvi.00555-26 (PMC13386995; doi:10.1128/jvi.00555-26)
Supplement: Supplemental figures — Fig. S1 and S2. [file jvi.00555-26-s0001.pdf]

Title:

Influenza A virus infection induces initial proliferation of commensal *Streptococcus pneumoniae* in the larynx leading to dissemination into the lower respiratory tract

Short title:

Laryngeal activation of commensal *S. pneumoniae* by influenza A virus

Authors:

Kohsuke Kato[1, 2, 3, 9], Keekushan Okamura[2, 9], Yuki Nakamura[2, 9], Mana Iwata[3], Mikako Hirohama[1], Yukino Ogura[1, 2, 3], Masamitsu Kono[4], Muneki Hotomi[4], Tomoko Sumitomo[5], Atsushi Kawaguchi[1, 2, 3, 6, 7, 8]*

[1] Department of Infection Biology, Institute of Medicine, University of Tsukuba, Tsukuba, Japan

[2] Graduate School of Comprehensive Human Sciences, University of Tsukuba, Tsukuba, Japan

[3] College of Medical Sciences, School of Medicine and Health Sciences, University of Tsukuba, Tsukuba, Japan

[4] Department of Otolaryngology-Head and Neck Surgery, Wakayama Medical University, Wakayama, Japan

[5] Department of Oral Microbiology, Graduate School of Biomedical Sciences, Tokushima University, Tokushima, Japan

[6] Transborder Medical Research Center, University of Tsukuba, Tsukuba, Japan

[7] Microbiology Research Center for Sustainability, University of Tsukuba, Tsukuba, Japan

[8] Center for Quantum and Information Life Sciences, University of Tsukuba, Tsukuba, Japan

[9] These authors equally contributed to this work

*Correspondence should be addressed to A. K. (email: ats-kawaguchi@md.tsukuba.ac.jp)

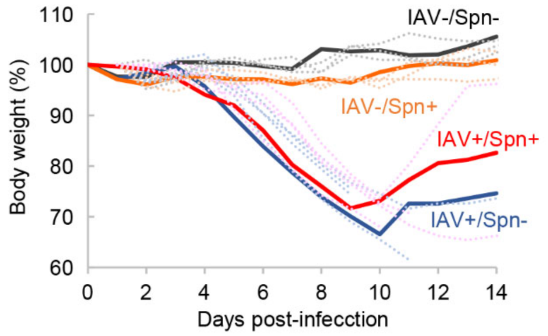

Supplementary Figure 1 Body weight changes in IAV-infected mice colonized with *S. pneumoniae*

Mice were divided into four groups based on IAV infection and *S. pneumoniae* (Spn) colonization status: uninfected and non-colonized (IAV-/Spn-; black), IAV-infected and non-colonized (IAV+/Spn-; blue), uninfected and Spn-colonized (IAV-/Spn+; orange), and IAV-infected and Spn-colonized (IAV+/Spn+; red). Body weight was monitored daily for 14 days post-infection. Solid lines represent the mean body weight per group, and dashed lines indicate individual data.

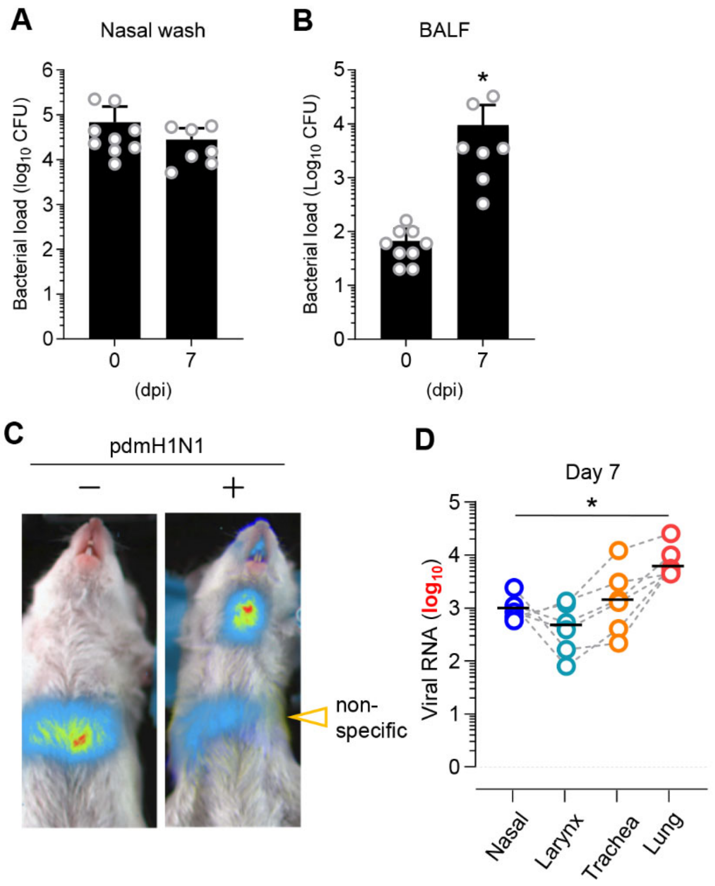

Supplementary Figure 2 Enhanced proliferation of colonizing pneumococci by pdmH1N1 infection

C57BL/6 mice colonized with EF3030 were intranasally infected with 15 µl of A/California/07/2009 (pdmH1N1) at a dose of 1 × 10^5 PFU. (A, B) Bacterial loads in nasal washes (A) and BALF (B) were examined at 0 and 7 days post-IAV infection (0 dpi, n = 9; 7 dpi, n = 7). *P < 0.05; two-tailed Student's t-test. Means ± SD are shown. (C) EF3030-AL-colonized C57BL/6 albino mice were intranasally infected with pdmH1N1. *In vivo* bioluminescence imaging was performed at 7 days post-IAV infection following intravenous injection of 30 mM AkaLumine-HCl in a 50 µl volume. A representative image from an infected EF3030-AL-colonized mouse is shown. (D) Viral loads in the nasal cavity, larynx, trachea, and lungs. Total RNAs were purified from the nasal cavity, larynx, trachea, and lungs of EF3030-colonized mice at 7 days post-pdmH1N1 infection (n = 4). The viral RNA levels were quantified using RT-qPCR. The results were normalized to the level of 18S rRNA. *P < 0.05; Friedman test followed by Dunn's multiple comparisons test. Means ± SD are shown.
